# P07 Toxicokinetic profile of VRP-034

**DOI:** 10.1093/jacamr/dlac004.006

**Published:** 2022-02-16

**Authors:** Yogeshkumar Vishnupant Murkunde, Saransh Chaudhary, Kamlesh Kumar Vishwakarma, Anmol Aggarwal, Rahul Chilbule

**Affiliations:** Venus Medicine Research Centre, India; Venus Medicine Research Centre, India; Venus Medicine Research Centre, India; Venus Medicine Research Centre, India; Venus Medicine Research Centre, India

## Abstract

**Background:**

Polymyxin B (PMB) can be an effective option for treating resistant Gram-negative pathogens, however, its use is severely compromised by its’ dose-limiting toxicity. VRP-034, a novel formulation of PMB, has previously reported a favourable safety profile compared with marketed PMB (Roy *et al.*^1^). This study was conducted to understand the toxicokinetic profile of VRP-034 vis-à-vis marketed PMB.

**Materials and methods:**

30 healthy Sprague-Dawley rats (divided into six groups of *n = *5) received one of three doses (3, 9 and 18 mg/kg/day) of either marketed PMB or VRP-034, administered subcutaneously q8 h over 2 days. Blood samples were collected prior to initiation of experiment and at 0.5, 1, 2, 4, 6, 8, 12 and 24 h after the last dose. Plasma concentrations of PMB were quantified by LC-MS. Additionally, renal toxicity was assessed via clinical pathology, kidney injury biomarkers, oxidative stress markers (3-nitrotyrosine and renal tissue lactate), gross and histopathologic observations of kidney. Urine and blood samples were collected at baseline and Day 2 for the assessment of kidney injury (KIM-1, cystatin-C, BUN and serum creatinine) using respective biochemical or ELISA kits. All values are expressed as mean ± SEM.

**Results:**

The plasma levels of PMB were found to increase in dose-dependent manner in both the groups. At 3 and 9 mg/kg/day, the plasma PMB exposure were similar for both VRP-034 and marketed PMB groups (AUC_0–24_: 6.47 ± 0.41 μg/mL·h versus 5.66 ± 0.24 μg/mL·h, *P>*0.05 and 22.90 ± 1.96 μg/mL·h versus 26.84 ± 1.43 μg/mL·h, *P>*0.05). At the highest dose of 18 mg/kg/day, the AUC_0–24_ was higher in the VRP-034 group (AUC_0–24_: 97.83 ± 12.95 μg/mL·h versus 70.48 ± 4.22 μg/mL·h), however this increase was not statistically significant (*P>*0.05). The levels of early kidney injury urinary biomarkers increased with dose in both the groups (Table 1). On Day 2, Kim-1 levels in the marketed PMB group increased from baseline by 3.96-fold (versus 2.31-fold in VRP-034 group), 8.21-fold (versus 5.41-fold) and 23.41-fold (versus 9.51-fold) at 3, 9, 18 mg/kg/day dose levels respectively. Likewise, cystatin-C levels increased by 6.22-fold (versus 3.26-fold in VRP-034 group), 6.25-fold (versus 3.67-fold) and 13.57-fold (versus 4.33-fold), respectively, at the three dose levels tested. Oxidative stress markers, tissue lactate and 3-nitrotyrosine, were significantly lower (*P<*0.05) for VRP-034 group compared with marketed PMB group at the highest dose. Histopathology revealed EGTI grade 3 kidney damage in the marketed PMB group compared with grade 1 damage in the VRP-034 group.

**Conclusions:**

VRP-034 exhibited a more promising toxicokinetic profile as compared with marketed PMB at all dose levels. Our future work will explore the clinical translation of these findings in humans.

**Table 1.** Kidney injury and oxidative stress markers for marketed PMB and VRO-034 groups

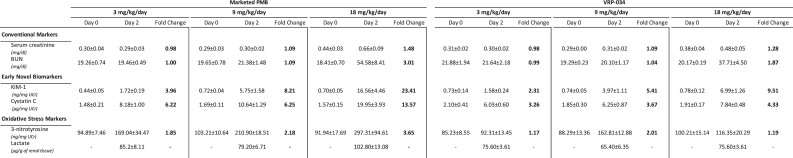

All values are reported as mean ± SEM.

Lactate marker was only measured on Day 2.

UCr, urine creatinine.
